# Dynamics of Known Long Non-Coding RNAs during the Maternal-to-Zygotic Transition in Rabbit

**DOI:** 10.3390/ani11123592

**Published:** 2021-12-19

**Authors:** Yu Shi, Mingcheng Cai, Kun Du, Xue Bai, Lipeng Tang, Xianbo Jia, Shiyi Chen, Jie Wang, Songjia Lai

**Affiliations:** 1 Farm Animal Genetic Resources Exploration and Innovation Key Laboratory Province, Sichuan Agricultural University, Chengdu 611130, China; 18227551690@163.com (Y.S.); dukun1672@163.com (K.D.); baixue333work@163.com (X.B.); soultang0329@163.com (L.T.); jaxb369@sicau.edu.cn (X.J.); chensysau@163.com (S.C.); wjie68@163.com (J.W.); 2 College of Landscape Architecture and Life Science/Institute of Special Plants, Chongqing University of Arts and Science, Yongchuan, Chongqing 402160, China; cmc527@163.com

**Keywords:** lncRNA, pre-implantation, maternal-to-zygotic transition, rabbit

## Abstract

**Simple Summary:**

We first revealed the expression profile of rabbit known lncRNAs during embryo pre-implantation development and showed minor and major wave of zygotic lncRNAs synthesis. The study then selected the differentially expressed (DE) lncRNAs between consecutive stages and predicted their potential target genes. The GO and KEGG analyses suggested that the lncRNAs participate in the regulation of embryo cleavage and development. Additionally, the sequential degradation of maternal lncRNAs showed that, like maternal mRNAs, maternal lncRNAs degradation occurred via maternal and zygotic pathways and the late-degraded lncRNAs might play a role in the degradation of mRNAs through mRNA surveillance pathway.

**Abstract:**

The control of pre-implantation development in mammals undergoes a maternal-to-zygotic transition (MZT) after fertilization. The transition involves maternal clearance and zygotic genome activation remodeling the terminal differentiated gamete to confer totipotency. In the study, we first determined the profile of long non-coding RNAs (lncRNAs) of mature rabbit oocyte, 2-cell, 4-cell, 8-cell, and morula embryos using RNA-seq. A total of 2673 known rabbit lncRNAs were identified. The lncRNAs exhibited dynamic expression patterns during pre-implantation development. Moreover, 107 differentially expressed lncRNAs (DE lncRNAs) were detected between mature oocyte and 2-cell embryo, while 419 DE lncRNAs were detected between 8-cell embryo and morula, consistent with the occurrence of minor and major zygotic genome activation (ZGA) wave of rabbit pre-implanted embryo. This study then predicted the potential target genes of DE lncRNAs based on the trans-regulation mechanism of lncRNAs. The GO and KEGG analyses showed that lncRNAs with stage-specific expression patterns promoted embryo cleavage and synchronic development by regulating gene transcription and translation, intracellular metabolism and organelle organization, and intercellular signaling transduction. The correlation analysis between mRNAs and lncRNAs identified that lncRNAs ENSOCUG00000034943 and ENSOCUG00000036338 may play a vital role in the late-period pre-implantation development by regulating *ILF2* gene. This study also found that the sequential degradation of maternal lncRNAs occurred through maternal and zygotic pathways. Furthermore, the function analysis of the late-degraded lncRNAs suggested that these lncRNAs may play a role in the mRNA degradation in embryos via mRNA surveillance pathway. Therefore, this work provides a global view of known lncRNAs in rabbit pre-implantation development and highlights the role of lncRNAs in embryogenesis regulation.

## 1. Introduction

Embryogenesis begins with mature oocyte and sperm fusing into one diploid genome. Massive reprogramming activities occur in multiple layers of gene expression regulation during pre-implantation development, switching control of embryo development from maternal factors to zygotic transcripts (maternal-to-zygotic transition, MZT). In the beginning, maternal RNAs and proteins exclusively guide the development while the zygotic genome remains quiescent. Subsequently, the zygotic genome is gradually activated to control the development (zygotic genome activation, ZGA). Notably, there are a few protein-coding RNAs in cells [1,2], most are non-coding RNAs that are essential in various cell activities [3,4].

Parents play a crucial role in the development of fertilized egg [5]. As a result, studies have assessed the underlying mechanism of zygotic nuclei in blastomere division and differentiation. Previous studies have revealed the expression patterns of transcripts from protein-coding genes through microarray analysis [6,7]. Advances in RNA sequencing technology, especially at the single-cell level, have helped reveal the whole scale of RNA profile, including mRNA and non-coding RNA. For instance, Tang et al. [8] found that miRNAs and mRNAs exhibited similar expression patterns in mouse pre-implantation development. Moreover, they showed that the inhibition of miRNA production in oocytes causes infertility or cleavage defects. The first signal of miRNAs synthesis can be determined as early as at 1-cell, followed by a rapid decrease of maternal miRNAs during the major ZGA in mouse embryogenesis [9]. Furthermore, X-link lncRNA Xist can mediate the X-inactivation in mouse and human embryos [10]. LncRNA H19-MBD1 complex combined with chromatin modifications can regulate imprint gene expressions [11]. The above studies suggest that non-coding RNAs play a vital role in embryogenesis regulation.

Long non-coding RNAs (lncRNAs) are longer than 200 nt [12,13,14]. lncRNAs can interact with multiple molecules, including DNA, RNA, and protein, for transcription modulating [15], epigenetic modifications [16,17], and translation [18]. Studies have shown that lncRNAs and mRNAs of pre-implanted mice [19] and human [20] embryos exhibit dynamic expression patterns during pre-implantation development. However, the understanding of the protein-coding genes expressed in rabbit pre-implantated embryos is limited, the identification and functional annotation of lncRNAs in this area are less understood.

Herein, the lncRNA expression profile of rabbit mature oocyte (represented by oocyte in the following description), 2-cell, 4-cell, 8-cell, and morula embryos was determined through RNA-Seq. The co-expression analysis between differentially expressed lncRNAs (DE lncRNAs) and mRNAs was conducted to elucidate the role of stage-enriched lncRNAs in rabbit pre-implantation development. The potential target protein-coding genes were further analyzed using Gene Ontology (GO) and Kyoto Encyclopedia of Genes and Genomes (KEGG) analyses. Moreover, the expression and degradation of maternal lncRNAs were analyzed.

## 2. Materials and Methods

### 2.1. Ethics Statement

All animal procedures were conducted according to the approved protocol of the Biological Studies Animal Care and Use Committee, Sichuan Province, China.

### 2.2. Animal Manipulation and Sample Collection

The study used 15 female Tianfu black rabbits (5–6 months old). The rabbits were caged separately and had free access to feed and water. After two-week acclimation, a hormone program of induction of follicle maturation and ovulation for the female rabbits was conducted as previously described in the lab [1]. Briefly, the rabbits were intramuscularly given with 70–80 IU of pregnant mare serum gonadotrophin (PMSG), then intravenously injected with 100 IU of human chorionic gonadotrophin (HCG; Ningbo Second Hormone Factory, Zhejiang, China) after 72 h. The female rabbits were then mated with the same healthy male rabbit. 

Rabbit embryos were collected according to the schedule and the morphologic characteristics as previously described [21]. Briefly, embryos were flushed out from the oviduct using 3% BSA in DPBS and washed thrice using DPBS. Oocytes or embryos were transferred into the modified acidic Tyrode (pH = 2.2) for 30 s, then cultured with 1% pronase for about 90 s until the zona pellucida and polar body were completely removed. Three oocytes or embryos from the same female rabbit were put into one collection tube.

### 2.3. RNA Sequencing and Data Processing

The RNA of each sample was extracted and amplified based on the Smart-Seq2 protocol [22]. The PCR products (1~2 kb) were purified and recovered using Ampure XP magnific beads (BECKMAN COULTER, Shanghai, China). The quality and concentration of PCR products were determined using Agilent 2100 High Sensitivity DNA Assay Kit (Agilent Technologies, Santa Clara, CA, USA) and Qubit 3.0 Flurometer (Life Technologies, Carlsbad, CA, USA), respectively. About 20 ng cDNA of a single sample was then sheared into about 300 bp fragments using ultrasound. The fragmented cDNA was end-repaired, dA-tailed, and adaptor ligated and then subjected to further PCR amplification. The final indexed PCR products were separated using 2% agarose gel electrophoresis and recovered using Gel Extraction Kit (CWBIO, Beijing, China). The library quality was assessed using Agilent 2100 bioanalyzer, and the concentration of each sample was determined using q-PCR (effective concentration > 2 nM). Sequencing was conducted using Illumina HiSeq 2500 platform (Illumina, CA, USA) generating 150 bp paired-end reads. 

### 2.4. Mapping, Filtering, and Quantification

Raw reads (FASTQ format) were filtered as follows: Trim Smart-seq2 public primer sequence from the reads (reads were discarded if the length of trimmed reads is lower than 30 bp); remove the contaminated reads for adapters (contaminated reads were defined when read bases contained more than 5 bp of adapter sequences); remove the low-quality reads (where the number of bases whose phred quality value was less than or equal to 19 accounted for more than 15%); remove the reads whose N bases were higher than 5% for total bases. Both ends of reads were removed if the above criteria characterized either end of read. The clean reads were then aligned to the rabbit reference genome OryCun2.0 (http://ftp.ensembl.org/pub/release-103/fasta/oryctolagus_cuniculus/dna/Oryctolagus_cuniculus.OryCun2.0.dna_sm.toplevel.fa.gz, accessed on 20 February 2021) together with the genome annotation OryCun2.0.103 (http://ftp.ensembl.org/pub/release-103/gtf/oryctolagus_cuniculus/Oryctolagus_cuniculus.OryCun2.0.103.gtf.gz, accessed on 20 February 2021) using HISAT2 2.1.0 [23]. Read counts for each lncRNA in each sample were counted using HTSeq 0.6.0. The quantified fragments per kilobase of transcript per million mapped reads (FPKM) were used to calculate the expression levels of each lncRNA. LncRNAs with FPKM ≥ 0.1 at least in one sample were considered effective lncRNAs.

### 2.5. Screening of DElncRNAs

Differential expression analysis was conducted using DESeq2 1.20.0 [24]. The lncRNAs between two consecutive stages showing |log2 fold change (FC)| ≥ 1 at adjust *p*-value < 0.05 were identified as significantly differentially expressed lncRNAs. Principal component analysis and K-means clustering analysis were performed using “FactoMiner” (https://cran.r-project.org/web/packages/FactoMineR/index.html, accessed on 12 November 2021) and “pheatmap” function (https://cran.r-project.org/web/packages/pheatmap/index.html, accessed on 12 November 2021) in R software. 

### 2.6. Co-Expresssion Analysis of mRNA and lncRNA 

Co-expression analysis between mRNA and lncRNA was performed (|Spearman correlation| ≥ 0.95) to investigate the potential role of lncRNAs in the rabbit pre-implantation development. The GO and KEGG analyses of the potential target genes were conducted using the online tool Database for Annotation, Visualization, and Integrated Discovery (DAVID 6.8, accessed on 12 November 2021) [25]. A *p*-value < 0.05 was considered as significantly enriched. Only part of these interactions between mRNA and lncRNA was drawn into the network diagram since there were too many pairs (|Spearman correlation| ≥ 0.98). 

### 2.7. Validation of DE lncRNAs Using RT-qPCR

Six DE lncRNAs, including two lncRNAs had the most frequent interactions with coding genes and four randomly selected DElncRNAs, were conducted qRT-PCR analysis to verify the validation of sequencing. Primers were designed using Primer-BLAST (https://www.ncbi.nlm.nih.gov/tools/primer-blast/index.cgi?LINK_LOC=BlastHome Accessed on 1 December 2021) and are listed in Table S1. The PCR reactions were performed on the CFX96TM Real-Time PCR Detection System (Bio-Rad, Hercules, CA, USA). PCR procedure was as follows: per-denaturation at 95 °C for 30 s, followed by 39 cycles of denaturation at 95 °C for 10 s, and annealing of each paired primers at corresponding temperature for 30 s. The no-template controls and negative controls without DNA polymerase were included in all qPCR runs. Amplifications were performed twice for each sample. Expression levels were normalized to GAPDH levels and calculated using the 2^−ΔΔCt^ method [26].

### 2.8. Statistical Analysis

The results of qRT-PCR were expressed as mean ± SEM. Statistical comparisons among groups were analyzed using graphpad Prism 6. A *p*-value < 0.05 was considered statistically significant.

## 3. Results

### 3.1. Temporal Expression Profile of lncRNA during Rabbit Pre-Implantation Development

**Figure 1:**
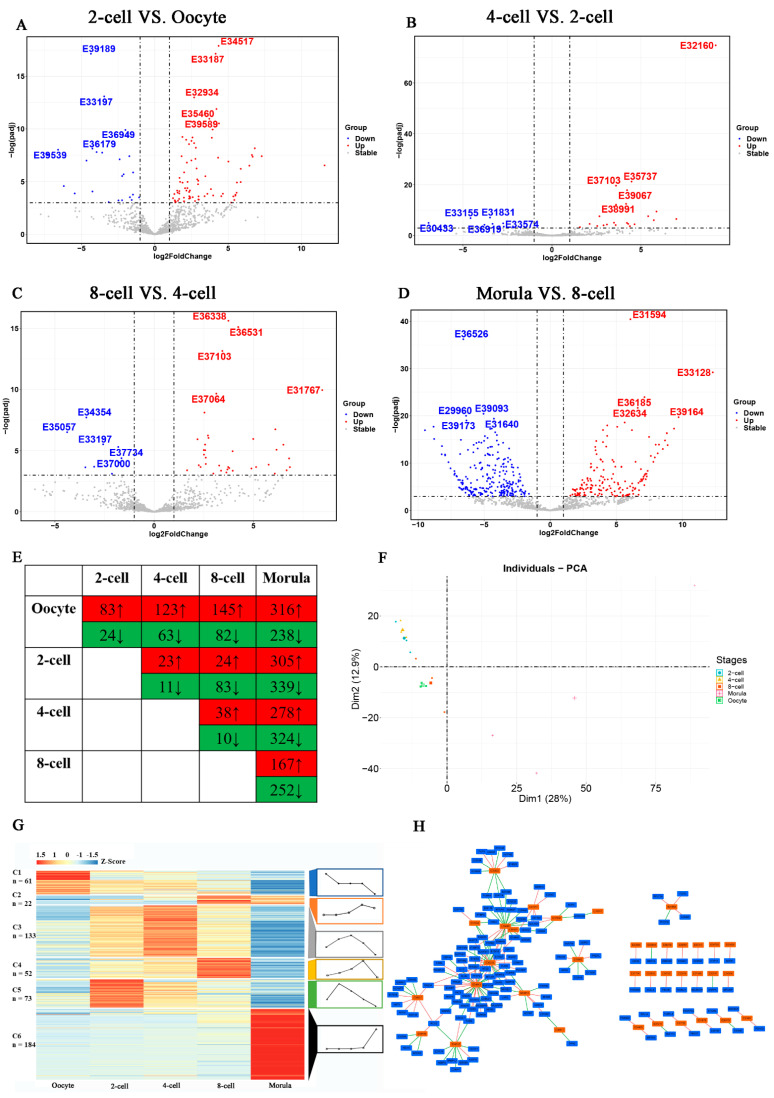
Dynamics of lncRNAs during rabbit pre-implantation development. (**A**–**D**) DE lncRNAs between consecutive stages: oocyte and 2-cell, 2-cell and 4-cell, 4-cell and 8-cell, 8-cell and morula; log(padj) (logarithm (adjust *p*-value)). Five lncRNAs up- or down-regulated with the lowest *p*-Value are labeled in the volcano plot, and their Ensembl ID ENSOCUG000000***** are abbreviated as E*****. For instance, E39189 represents ENSOCUG00000039189. (**E**) Number of DE lncRNAs between consecutive stages. (**F**) Principal component analysis of all identified lncRNAs; PCA (Principal component analysis); Dim1: (dimension1); Dim2 (dimension2). (**G**) Expression changes of DE lncRNAs analyzed using the k (clustering method); C (cluster). (**H**) Network diagram of co-expression analysis between lncRNAs and mRNAs (|Spearman correlation| ≥ 0.98).

A total of 2637 known rabbit lncRNAs were identified. The lncRNAs exhibited dynamic expression patterns during rabbit pre-implantation development. The up- and down-regulated lncRNAs were screened from the paired comparison of consecutive stages (Figure 1A–E). A total of 83 and 24 lncRNAs were significantly upregulated and downregulated, respectively, between oocytes and 2-cell embryos. A total of 23 and 11 lncRNAs were significantly up- and downregulated, respectively, between 2-cell and 4-cell embryos. Only 38 and 10 lncRNAs were significantly up- and downregulated, respectively, between 4-cell and 8-cell embryos. lncRNAs expression was significantly altered during the development from 8-cell to morula, having 419 significantly differential expressed lncRNAs (167 up-regulated and 252 down-regulated). Similarly, principal component analysis (PCA) showed slight lncRNAs expression changes between various consecutive stages: oocyte and 2-cell embryo, 2-cell and 4-cell embryo, and 4-cell and 8-cell embryo, and significant changes between 8-cell and morula embryo (Figure 1F). 

The DE lncRNAs was sorted into 6 clusters using K-means clustering approach to determine the correlation of DE lncRNAs and rabbit pre-implantation development (Figure 1G). Most lncRNAs were in cluster C6 and had a relatively stable and low expression level before morula stage, suggesting a considerable group of lncRNAs were synthesized at morula stage. Similarly, lncRNAs in cluster C2 had relatively high expression level at the late period of embryo pre-implantation development. The expression levels of lncRNAs in cluster C1 gradually decreased from oocyte to morula, implying that these lncRNAs may be maternal original. Unlike clusters C1, C2, and C6, the expression levels of lncRNAs in clusters C3, C4, and C5 increased after fertilization, peaking at the mid-period of pre-implantation development, then decreasing to the lowest level in morula embryo.

**Figure 2:**
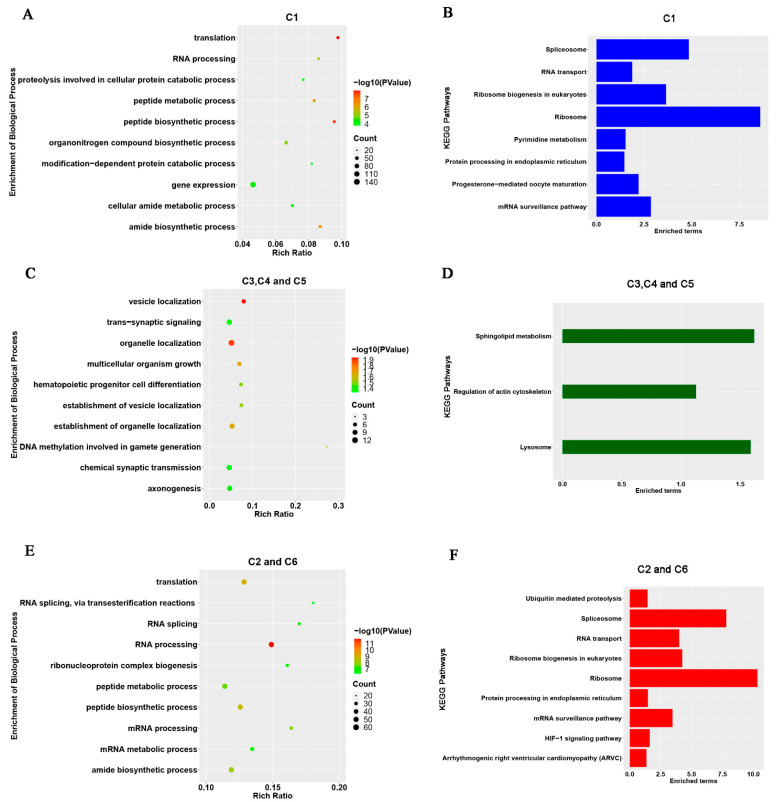
GO and KEGG analyses of DE lncRNA. (**A**,**C**,**E**) The top 10 significantly enriched terms of biological process (*p* < 0.05). (**B**,**D**,**F**). The top 10 significantly enriched KEGG pathways (*p* < 0.05). C1–C6: (cluster 1 to cluster 6) identified by k (clustering method from previous analysis). GO (Gene Ontology). KEGG (Kyoto Encyclopedia of Genes and Genomes).

The potential target protein-coding genes were predicted by analyzing the correlation between DE lncRNAs and mRNAs expression levels to assess the functions of lncRNAs in rabbit pre-implantation development comprehensively. A total of 4091 highly correlated pairs were detected between DE lncRNAs and protein-coding genes (|Spearman correlation| ≥ 0.95, Table S2). The network diagram was then drawn using some of the correlated pairs (|Spearman correlation| ≥ 0.98, Figure 1G). The network showed that lncRNAs ENSOCUG00000034943 and ENSOCUG00000036338 had the most frequent interactions with coding genes, among which Interleukin Enhancer Binding Factor 2 (*ILF2*) was positively regulated by the two lncRNAs. This study then sorted all the target genes (|Spearman correlation| ≥ 0.95) into three subgroups based on the expression pattern of the corresponding lncRNAs: maternal group (lncRNAs decreased from oocyte to morula), maternal-to-zygotic group (C3–C5, lncRNAs showed relative high expression levels in the mid-period of pre-implantation development), and zygotic group (C2 and C6, lncRNAs showed relatively high expression levels in the late period of pre-implantation development). The top 10 significantly enriched terms in biological process (BP) are shown in Figure 2A–C (*p* < 0.05). The terms enriched in the maternal group (C1 cluster) were mainly involved in the establishment of gene transcription and translation (translation, RNA processing, peptide metabolic/biosynthesis process, gene expression, cellular amide metabolic/biosynthetic process) and material preparation for cell divisions (proteolysis involved in cellular protein catabolic process, organonitrogen compound biosynthetic process, and modification-dependent protein catabolic process). The enriched terms in maternal-to-zygotic group (C3–C5 clusters) were mainly associated with multicellular development of the embryo (multicellular organism growth, DNA methylation involved in gamete generation, hematopoietic progenitor cell differentiation, axonogenesis) and signaling transduction among cells (trans-synaptic signaling, trans-synaptic-signaling and chemical synaptic transmission). Notably, most enriched terms in the zygotic group (C2 and C6 clusters) were related to post-transcriptional processes (translation, RNA processing, peptide biosynthetic/metabolic process, amide biosynthetic process). The KEGG analysis results showed that the top 3 enriched pathways in the maternal and zygotic groups were spliceosome, RNA transport, and ribosome (*p* < 0.05). The top 3 enriched pathways in maternal-to-zygotic group were the regulation of actin cytoskeleton, lysosome, and sphingolipid metabolism (*p* < 0.05).

### 3.2. Maternal lncRNAs Degradation

**Figure 3:**
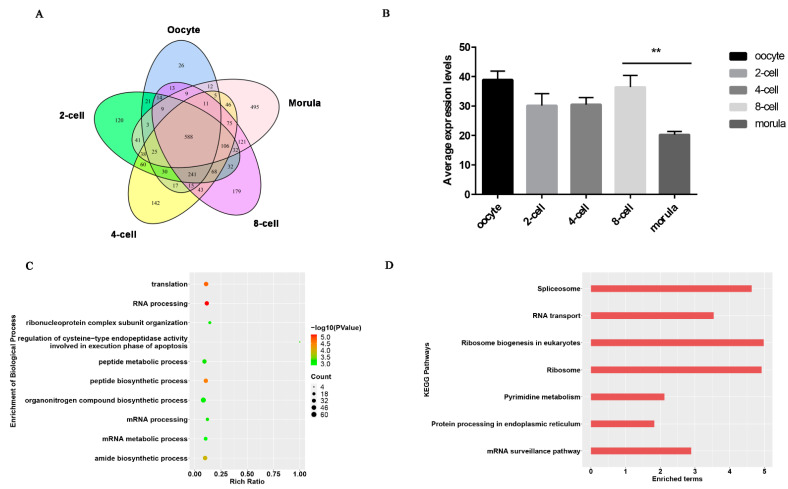
Analysis of maternal lncRNAs. (**A**) Numbers of lncRNAs expressed in each stage. (**B**) Average expression levels of 588 maternal lncRNAs; **—indicates statistically significant with *p* < 0.01. (**C**,**D**) GO and KEGG analyses of maternal lncRNAs (*p* < 0.05).

Maternal clearance is one of the major molecular activities in pre-implantation embryos. This study further analyzed the expression changes of maternal lncRNAs to assess if maternal lncRNAs undergo extensive degradation during MZT. The number of expressed lncRNAs at each stage was counted and analyzed. Each stage had some exclusively expressed lncRNAs (Figure 3A). Approximately 500 lncRNAs were exclusively expressed in morula, more than the sum of that in the four first stages, suggesting that abundant zygotic lncRNAs were transcribed in morula embryos. Interestingly, up to 588 lncRNA, half of the maternal lncRNAs, showed expression across the five stages. The study then analyzed the average expression of these lncRNAs and found that these lncRNAs had relatively stable expression levels in the four first stages and significantly decreased the expression levels at morula stage (Figure 3B, *p* < 0.01). The study also predicted the potential target genes of these late-degraded lncRNAs (|Spearman correlation| ≥ 0.95, Table S3). The top 10 significantly enriched terms were highly related to translation, RNA processing, peptide metabolic/biosynthesis process, organonitrogen compound biosynthetic process, amide biosynthetic process, etc., (*p* < 0.05). KEGG analysis showed that these lncRNAs were mainly involved in pathways of spliceosome, RNA transport, ribosome, pyrimidine metabolism, protein processing in endoplasmic reticulum, and mRNA surveillance pathway (Figure 3C,D, *p* < 0.05).

### 3.3. Validation of DE lncRNAs by qRT-PCR

**Figure 4:**
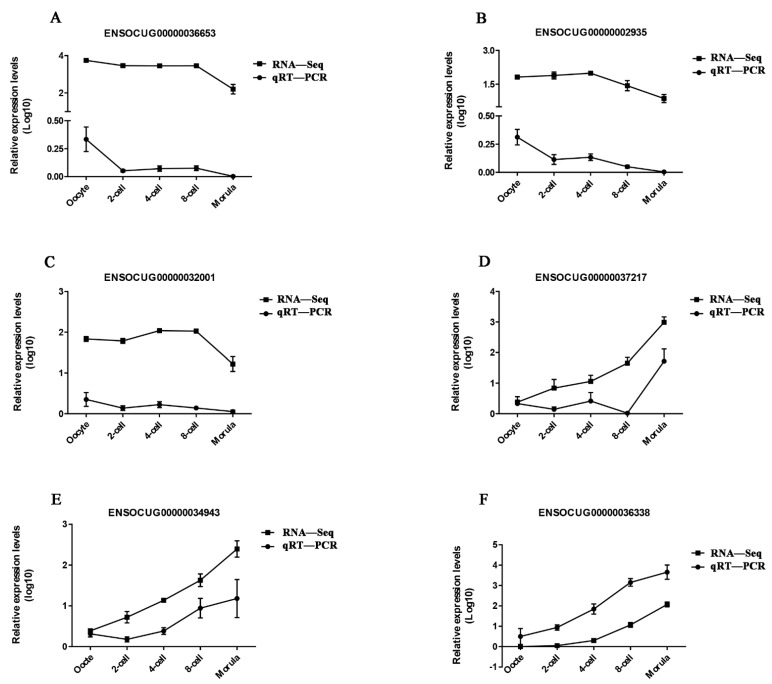
Validation of six DE lncRNAs including ENSOCUG0000036653 (**A**), ENSOCUG0000002935 (**B**), ENSOCUG0000032001 (**C**), ENSOCUG0000037217 (**D**), ENSOCUG0000034943 (**E**) and ENSOCUG0000036338 (**F**) by RT-qPCR.

The expression levels of four DE lncRNAs (ENSOCUG00000036653, ENSOCUG00000002935, ENSOCUG00000032001 and ENSOCUG00000037217) were randomly selected and the top 2 lncRNAs with the most frequent interactions with mRNAs (ENSOCUG00000034943 and ENSOCUG00000036338) were validated using qRT-PCR. The changes of selected lncRNAs determined by qRT-PCR were similar to those quantified using RNA-Seq regardless of the differences in the magnitude of fold-changes, indicating that our RNA-Seq profile was reliable and effective (Figure 4).

## 4. Discussion

The recovery of pluripotency is the most important pre-requisite for the fertilized egg to become a new life with multiple differentiated tissues and organisms. LncRNAs can coordinate with proteins involved in pluripotency and differentiation of embryonic stem cells [27]. Several lncRNAs has been identified associated with imprint gene expression [10,11,28] of embryo. Moreover, studies in recent years have revealed and analyzed the dynamic expression patterns of the lncRNAs identified during embryo pre-implantation development. For instance, Zhang et al. [29] identified 5563 novel lncRNAs in mouse cleavage stage embryos. In addition, more than half of known human lncRNAs have been detected in 90 human embryonic cells with stage-specific expression patterns [20], indicating that lncRNAs might play a role in the recovery of pluripotency for embryo. Meanwhile, several studies have identified many lncRNAs in various rabbit tissues, which are suggested to regulate the growth and development of rabbit adipose tissue [30], skeletal muscle [31], and hair follicle [32]. This study detected 2637 known rabbit lncRNAs were with dynamic changes during rabbit pre-implantation development. Previous research has indicated that the major ZGA of rabbit occurs between 8- and 16-cell embryos [33], implying that a large amount of zygotic synthesized transcripts can be identified during this period. This study identified 419 DE lncRNAs between 8-cell and morula, accounting for about 70% of the total DE lncRNAs, suggesting that major changes of lncRNAs profile occur during this period. Besides, 107 DE lncRNAs were detected between oocyte and 2-cell embryo of which 83 lncRNAs were up-regulated in 2-cell, possibly due to the minor ZGA wave in 1-cell rabbit embryo [34]. The above analysis indicates that the dynamic of lncRNA in rabbit embryo was consistent with the ZGA process.

LncRNAs have various functions, including cis- or trans- regulation of gene transcription, chromatin organization, and interaction of proteins or RNAs [35]. Previous research has shown lncRNA profile in vivo and in SCNT mouse pre-implantation embryos. Most maternal lncRNA is degraded after ZGA during mouse in vivo embryo development, while this phenomenon has not been observed in nuclear transfer embryos, implying that the accurate lncRNAs reprogramming is essential for normal pre-implantation embryo development [36]. Herein, the expression of LncRNAs in cluster C1 gradually decreased with embryo cleavage, suggesting that these lncRNAs might play a role in oogenesis and oocyte maturation, but have detrimental effects on embryo development. The potential target genes of lncRNAs in C1 were mainly involved in gene transcription, RNA splicing, and organization of cellular component, emphasizing the role of maternal molecules in the process of early embryo cleavage and conferring another important function that activates the embryonic transcripts synthesis [37,38]. However, about 40% of DE lncRNAs were in clusters C2 and C6, and their expression rapidly and significantly increased at the late period of pre-implantation development. Furthermore, their potential target genes were mainly enriched in various BP, including RNA processing and protein synthesis, suggesting that lncRNAs highly expressed between 8-cell and morula stage prepare for future blastocyst development and further differentiation. LncRNAs in C3–C5 clusters showed comparably higher expression levels between 2- and 8-cell stages when fertilized egg had cleaved, and synchronous development among blastomere required frequent and timely signal exchange. Nevertheless, the expression of these lncRNAs immediately decreased after they reached the peak level. This wave-like expression pattern has also been observed in the profile of protein-coding gene of mouse [6] and human embryos [20], implying specific lncRNAs are needed in distinct stages, and there may be a negative feedback mechanism suppressing their transcription.

The co-expression analysis showed that *Ilf2* gene was positively correlated with lncRNAs ENSOCUG00000034943 and ENSOCUG00000036338, which were all in cluster C6. Studies have indicated that *IlfF2* is involved in RNA splicing and DNA damage resistance [39]. The down-regulated lncRNA HCP5 in the human premature ovarian insufficiency (POI) model can partially cause dysfunction of granulosa cells due to impaired DNA damage repair mediated by Y-box binding protein 1 and ILF2 [40]. A previous experiment showed that the deletion of long intergenic RNA (linc-GET) is associated with the developmental arrest of mouse 2-cell embryo. Linc-GET can inhibit abnormal splicing of cyclin-dependent kinase 1 (CDK1), the key G2 to M transition-associated gene, by downregulating the expression of several proteins, including ILF2. Interestingly, this study detected the linc-GET expression only in mouse 2-cell and 4-cell embryos, implying that linc-GET can specifically prevent mouse 2-cell block [41]. Notably, no such block was observed in rabbit embryo. The expression levels of the two lncRNAs and *Ilf2* gene gradually increased with the development of rabbit embryos, implying that lncRNAs ENSOCUG00000034943 and ENSOCUG00000036338 may be involved in the late period of rabbit embryo development by promoting *Ilf2* gene expression. Maternal transcripts accumulate in oocytes during oogenesis and exclusively guide the meiotic maturation and early embryo development after fertilization. Selective degradation of mRNAs is necessary for embryogenesis. Moreover, rapidly degradable mRNAs may negatively affect embryo development [42]. Maternal mRNAs degradation in animals is promoted by two factors: maternal factors in mature oocyte and zygotic genomic molecules [43,44]. This study found that half of the maternal lncRNAs had stable expression at the first four stages but down-regulated the expression at morula stage, implying that maternal and zygotic factors are involved in the degradation of maternal lncRNAs. The potential target genes of the late-degraded lncRNAs were enriched in the RNA transport, processing, and mRNA surveillance pathways. The mRNA surveillance pathway regulates mRNA degradation, implying that maternal lncRNAs may be involved in regulating mRNA degradation in pre-implanted embryos. Mammalian ovaries are characterized by cyclic follicular maturation, ovulation, and resorption of corpus luteum throughout female reproductive life span, which are regulated by autocrine, paracrine, juxtacrine, and endocrine factors [45]. Follicle stimulation hormone (FSH) and luteinizing hormone (LH), paracrine cues, are key regulators for recruiting dominant follicles and inducing ovulation [46]. Pregnant mare serum gonadotropin (PMSG) and HCG preparations for either estrus synchronization or superovulation are increasingly used for the endocrinologic or reproductive experiment [47,48]. However, the effect of gonadotrophic stimulation on female reproductive performance and fertilized egg should be assessed. Although several experiments have been conducted using species to assess the above, they have contradictory conclusions. For instance, a study showed that combined PMSG and HCG did not alter the fertility ability of ovulated egg and the proportion of offspring obtained from 2-cell embryo transfer in rats [48]. While other study concluded that the ability of fertilized oocyte developing to embryo decreased after multiple gonadotropic stimulation [49]. Embryo development is mainly characterized by epigenetic re-programming. Gonadotropic stimulation can downregulate the acetylation level of histone 4 at lysine 12 (H4K12ac) in the early embryos [49]. Although superovulation causes average global DNA methylation changes of embryos in mice, additional experiment is needed to verify the result since they did not have repetitive samples [50]. In addition, superovulation only affected the methylation levels of several imprinted genes in the blastocyst of mouse [51]. But no defects of DNA acquisition of imprint gene in oocyte were observed [52]. No significant differences of imprint genes *SNRPN*, *H19*, *Kcnq1ot1* of embryos were observed between spontaneously ovulated or superovulation group. Although superovulation up-regulated imprint gene *LGF2* at each stage, the expression patterns of *LGF2* during pre-implantation development of embryos were consistent with the spontaneously ovulated group [53]. Notably, superovulation is commonly used to obtain enough samples, especially for transcriptomic or genomic analysis, due to limited oocyte and embryo samples [19,54]. Herein, hormone treatment was used to conduct superovulation, and the lncRNA profile was analyzed from a vertical aspect in the study, which should represent the lncRNA dynamics of rabbit pre-implantation embryos. However, further studies should explore whether hormone treatment can lead to a whole scale lncRNA alterations and its potential effects on rabbit embryo development.

## 5. Conclusions

This study detected 2637 known rabbit lncRNAs with stage-specific expression patterns. lncRNAs expression was significantly altered between consecutive stages, especially between oocyte and 2-cell embryos, and 8-cell and morula embryos, consistent with the time of the minor and major wave of ZGA in rabbit, respectively. A total of 4091 highly correlated pairs between mRNA and DE lncRNAs were identified based on the trans-regulation mechanism of lncRNA. The following functional analysis indicated that the stage-enriched lncRNAs promote embryo cleavage and synchronic development by regulating gene transcription and translation, intracellular metabolism and organelle organization, and intercellular signaling transduction. Two lncRNAs ENSOCUG00000034943 and ENSOCUG00000036338 may play a role in the late period of rabbit embryo development by promoting *Ilf2* gene expression. Like maternal mRNA degradation in embryos, the degradation of maternal lncRNAs occurred via both maternal and zygotic pathways. Furthermore, the function analysis of the late-degraded lncRNAs suggested that these lncRNAs were involved in mRNA surveillance pathway, indicating that these lncRNAs may have a role in mRNA degradation in embryos.

## Data Availability

The datasets generated for this study has been deposited in the Sequence Read Archive (https://www.ncbi.nlm.nih.gov/sra, accession Number PRJNA785143, accessed on 4 December 2021) at NCBI, with the BioSample ID: SAMN23532251- SAMN23532265.

## Supplementary Materials

The following are available online at https://www.mdpi.com/article/10.3390/ani11123592/s1, Table S1: Primers used for RT-qPCR in this study. Table S2: Pearson correlation coefficient between all DE lncRNAs and mRNAs. Table S3: Pearson correlation coefficient between late-degraded maternal lncRNAs and mRNAs.
