# Supplementary material for: Dynamics of Known Long Non-Coding RNAs during the Maternal-to-Zygotic Transition in Rabbit
Source: Animals (Basel). 2021 Dec 19;11(12):3592. doi: 10.3390/ani11123592 (PMC8698111; doi:10.3390/ani11123592)
Supplement: Supplementary file 1 [file animals-11-03592-s001.zip › Supplementary Table S1.pdf]

Table S1. Primers of genes used in RT-qPCR.

Gene ID	Primer sequence	Product length (bp)
GAPDH	F: CTTCGGCATTGTGGAGGG	130
	R: GGAGGCAGGGATGATGTTCT	
ENSOCUG00000036653	F: GTGGTGCCTACCCTCTTGAC	97
	R: TATCTCGCTTGCCTTGGGTG	
ENSOCUG00000002935	F: GCACGATGTCCTTCAGGGTT	184
	R: GCTTCCCTGACCAGTACGTC	
ENSOCUG00000032001	F: GGAGACCCACAAACTGCGTA	109
	R: TAAGGACACTGAACCACGGC	
ENSOCUG00000037217	F: TGTTTCCGTCTCTGGGTTCC	148
	R: CCCAGGACATTCCCCAATCC	
ENSOCUG00000034943	F: AGTGTTGAGCGTGGGAAGAT	104
	R: ACACACAGAAGCCTGAGGTC	
ENSOCUG00000036338	F: TGCTGGTTTGGGTTTGACTTG	80
	R: GGTTCCACTTGAATGCAGGGTA	
